# Supplementary figures and images for: Unveiling the Relationship between Ceftobiprole and High-Molecular-Mass (HMM) Penicillin-Binding Proteins (PBPs) in Enterococcus faecalis
Source: Antibiotics (Basel). 2024 Jan 9;13(1):65. doi: 10.3390/antibiotics13010065 (PMC10812503; doi:10.3390/antibiotics13010065)

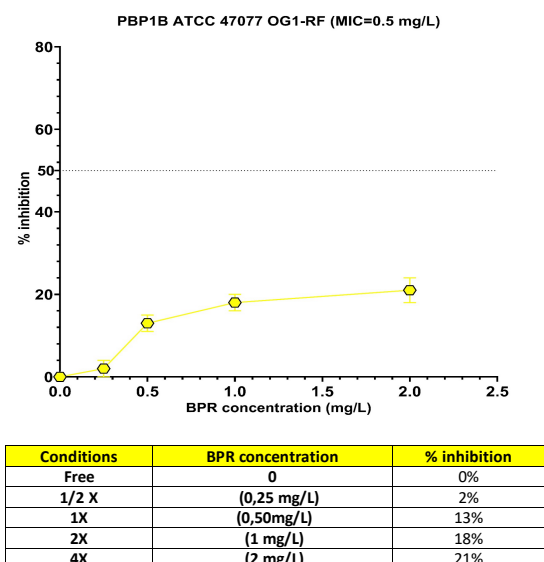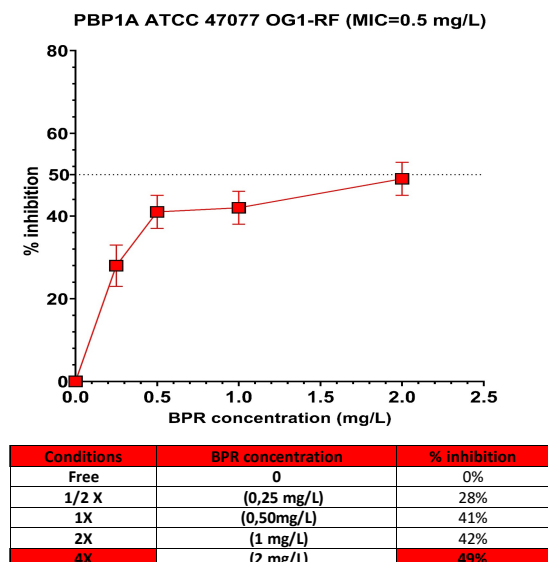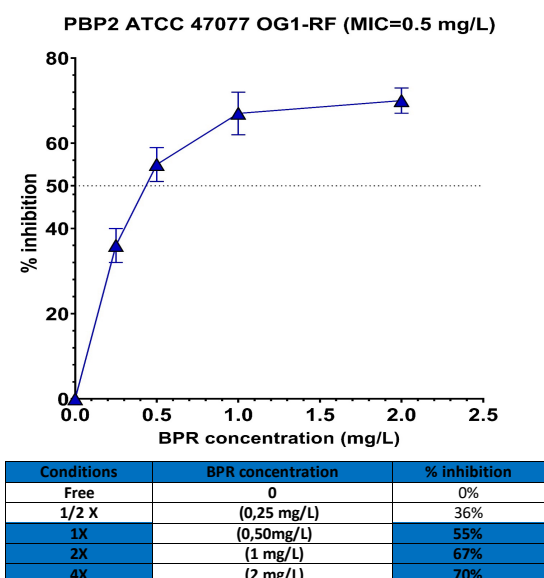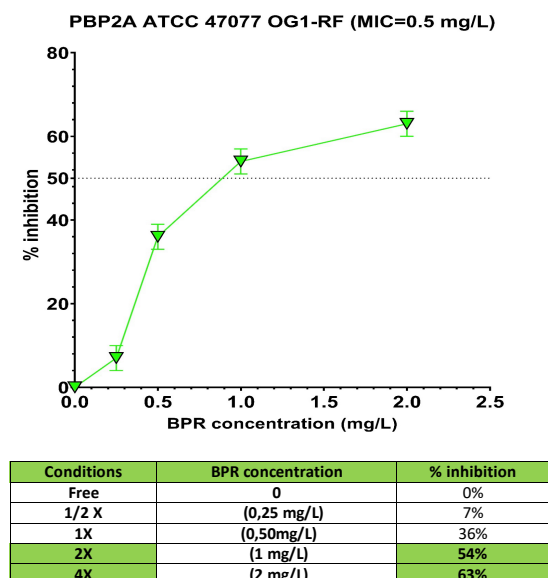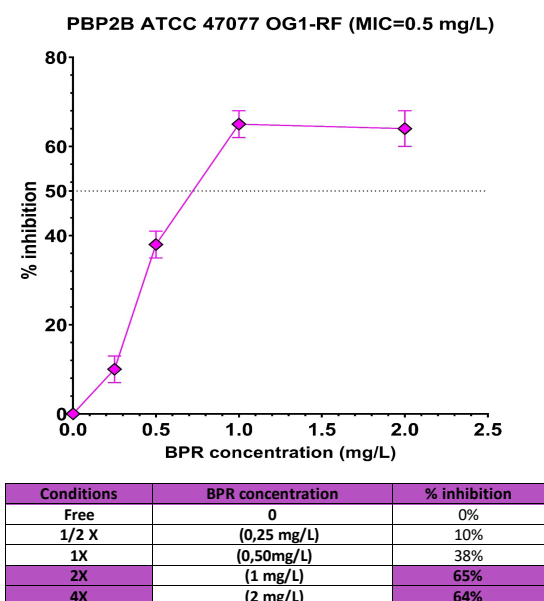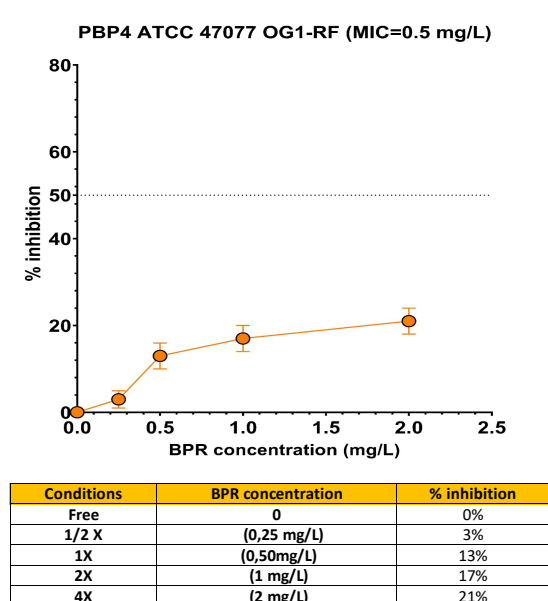

Figure S1. ATCC 47077 detailed PBPs inhibition rates.

Supplement: Supplementary file 1 [file antibiotics-13-00065-s001.zip › Supplementary material. Figure S1.pdf]

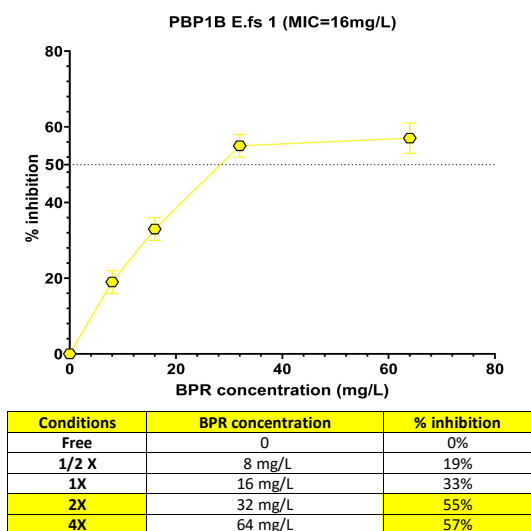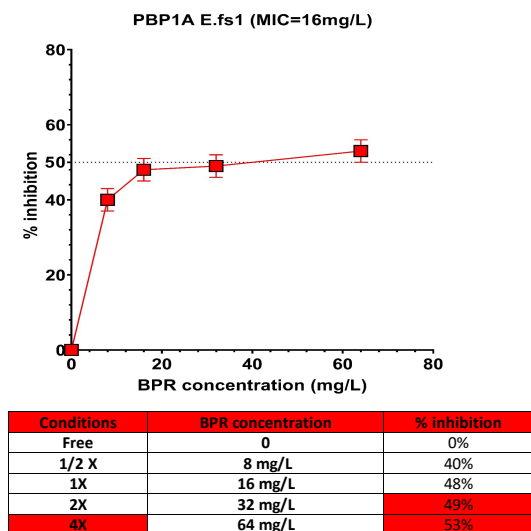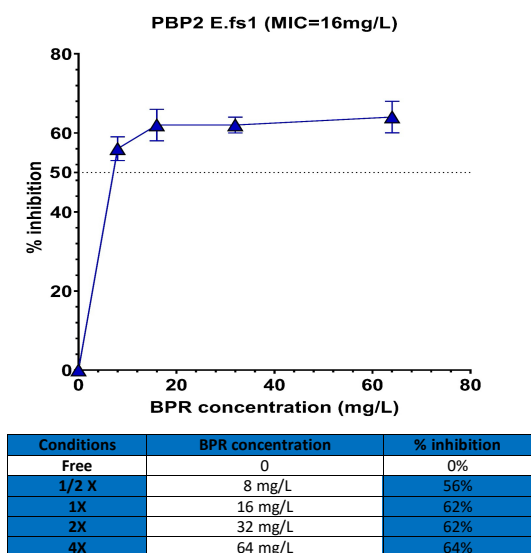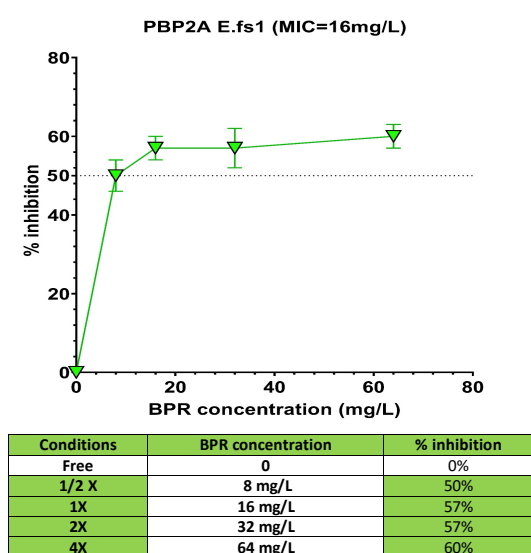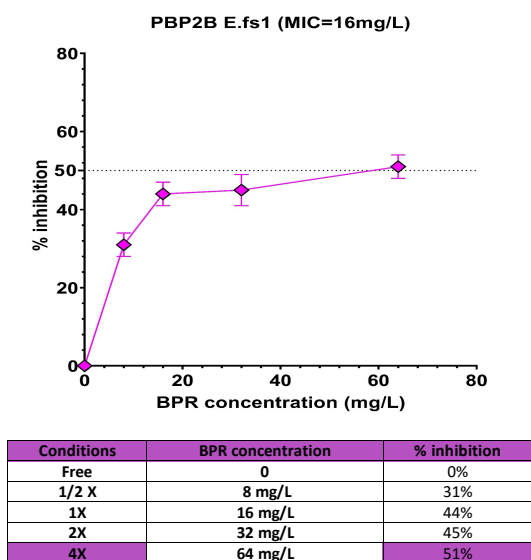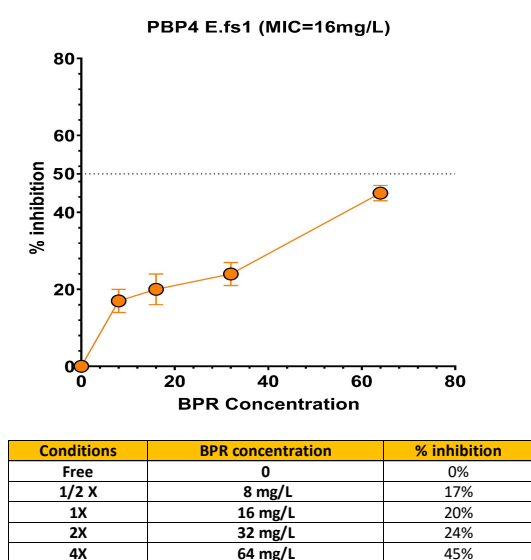

Figure S2. E.fs1 detailed PBPs inhibition rates.

Supplement: Supplementary file 1 [file antibiotics-13-00065-s001.zip › Supplementary material. Figure S2.pdf]

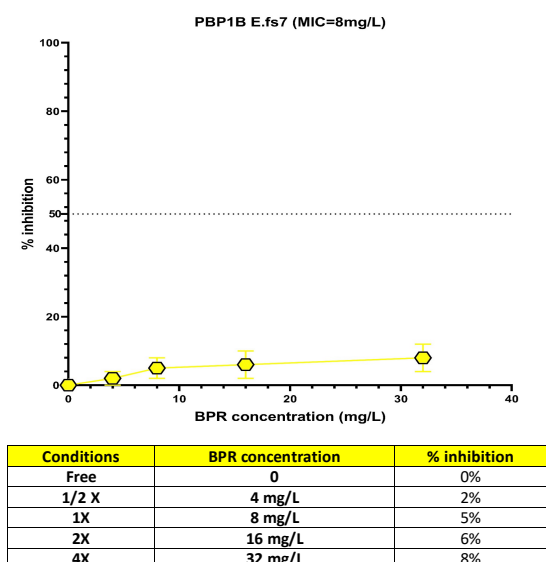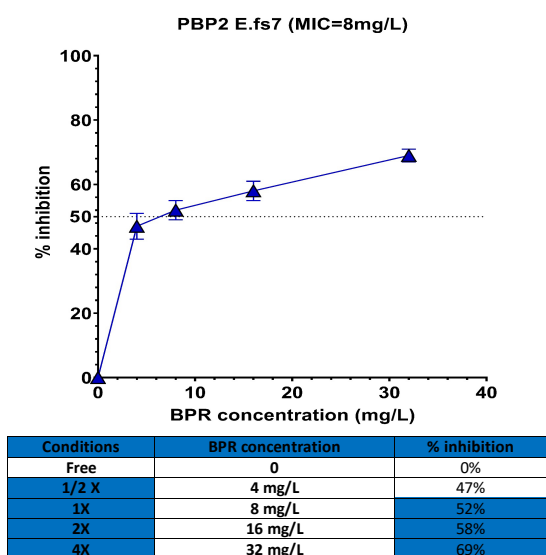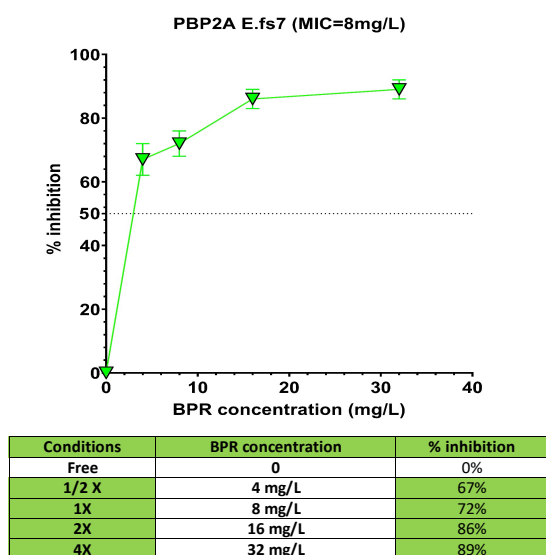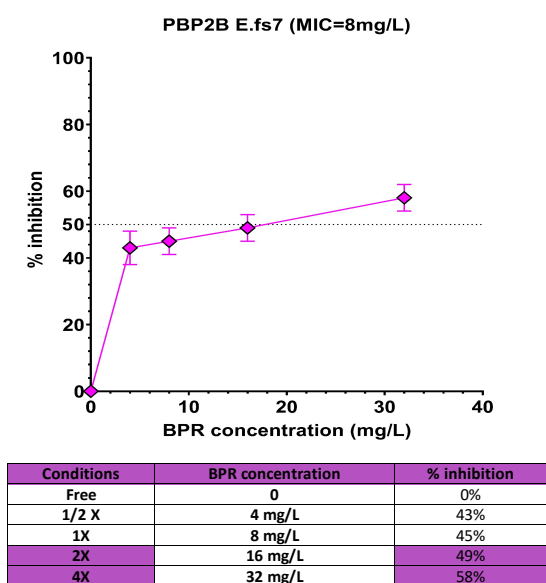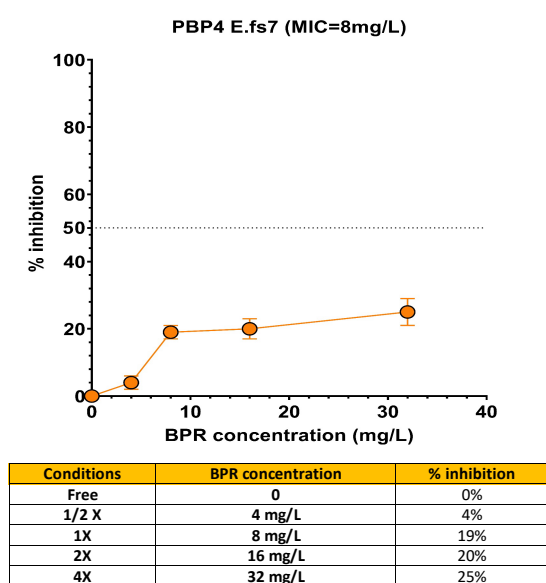

Figure S3. E.fs7 detailed PBPs inhibition rates.

Supplement: Supplementary file 1 [file antibiotics-13-00065-s001.zip › Supplementary material. Figure S3.pdf]

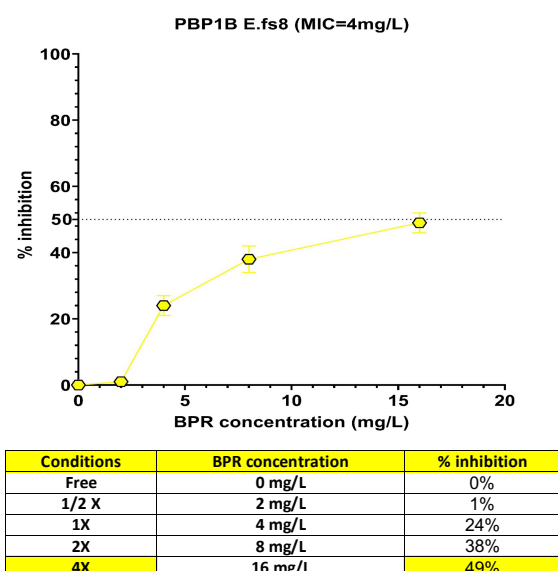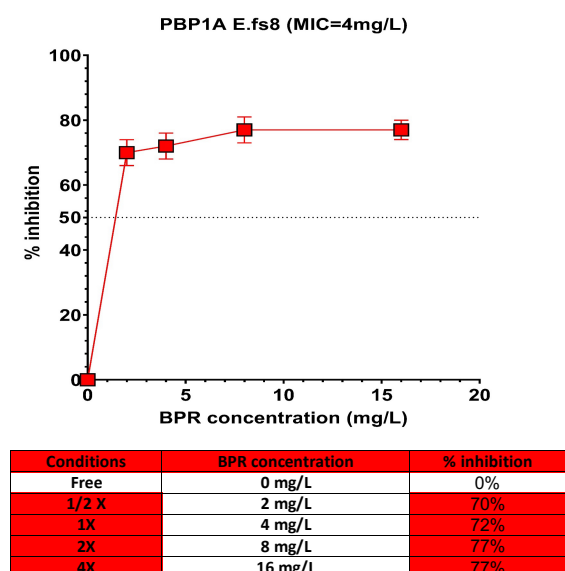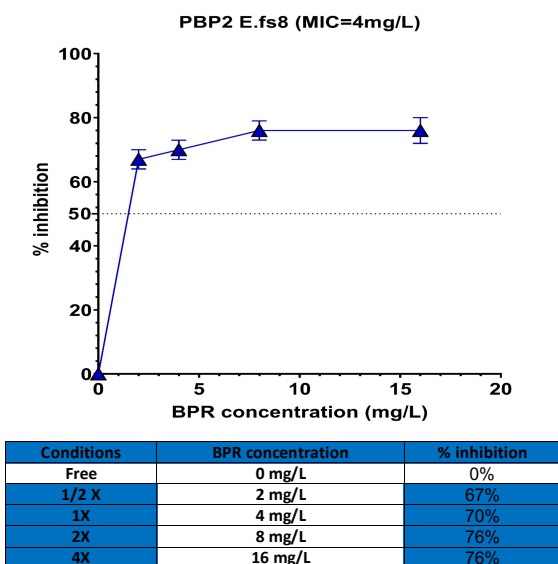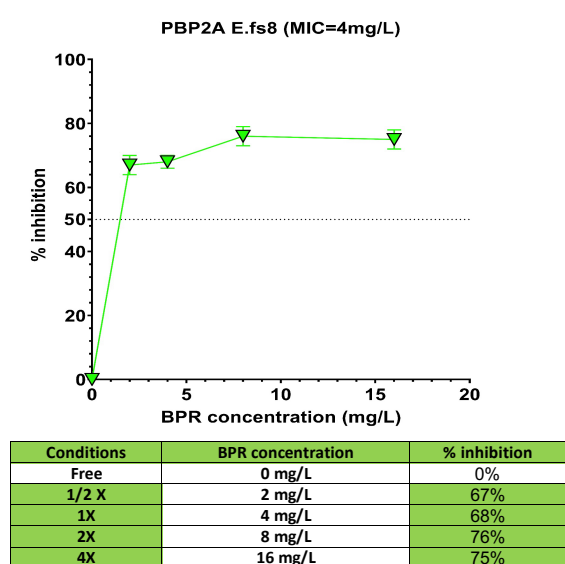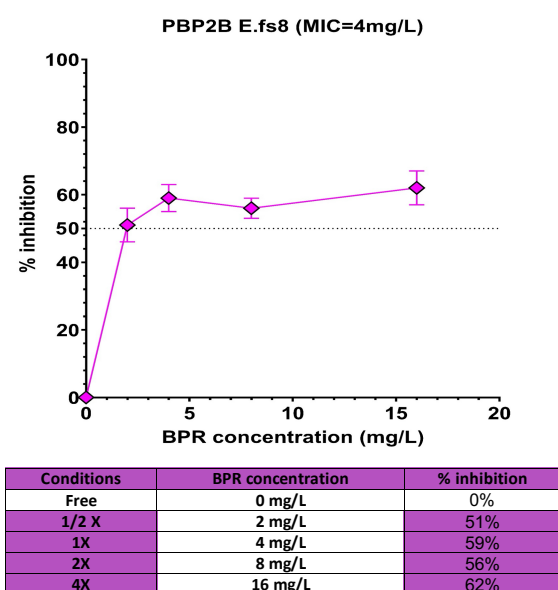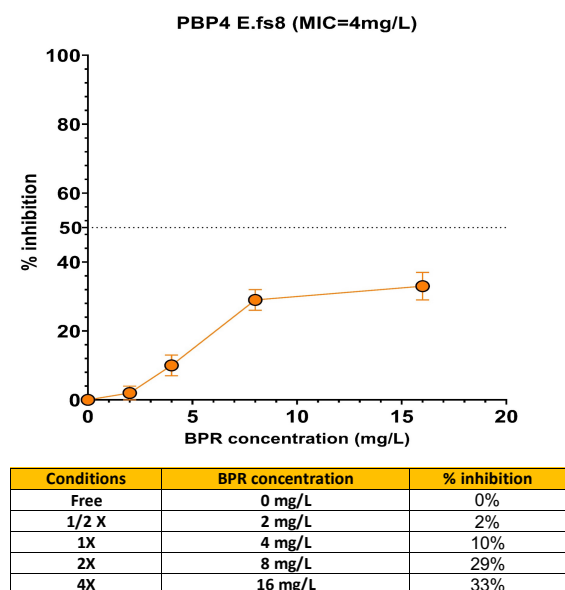

Figure S4. E.fs8 detailed PBPs inhibition rates.

Supplement: Supplementary file 1 [file antibiotics-13-00065-s001.zip › Supplementary material. Figure S4.pdf]

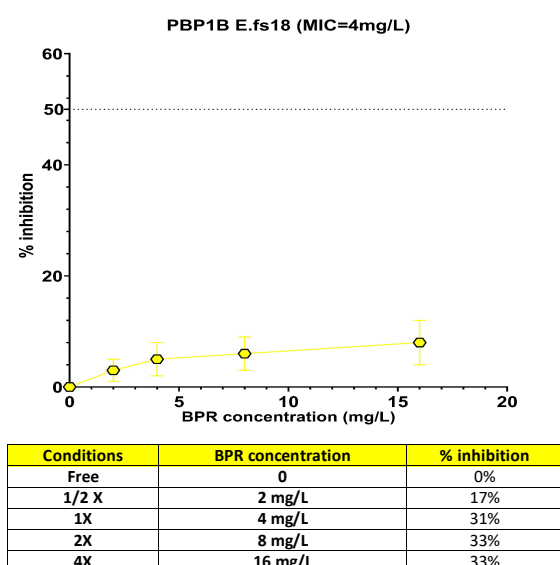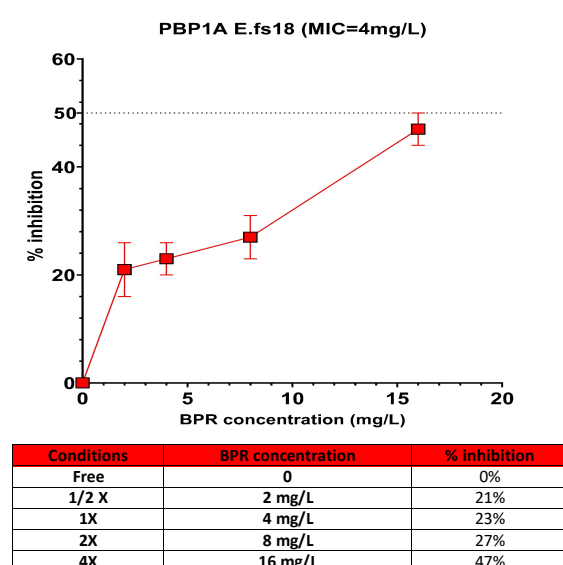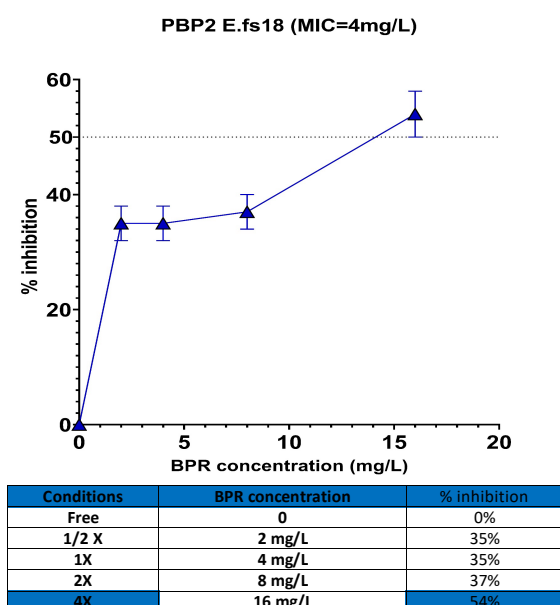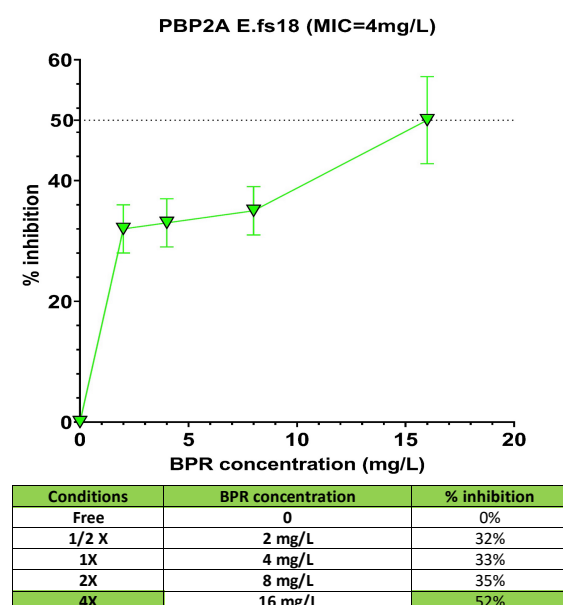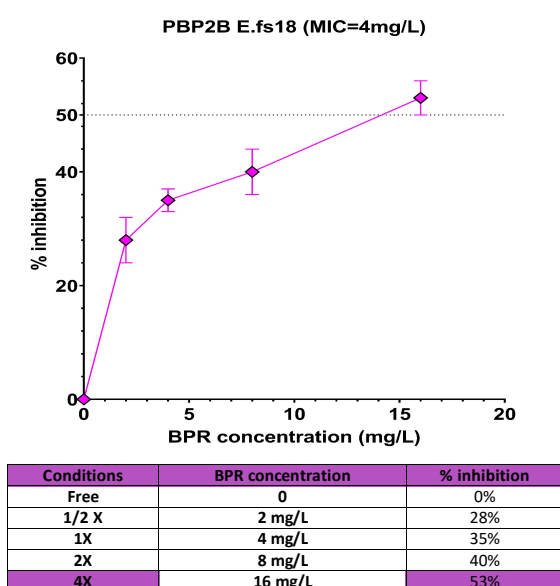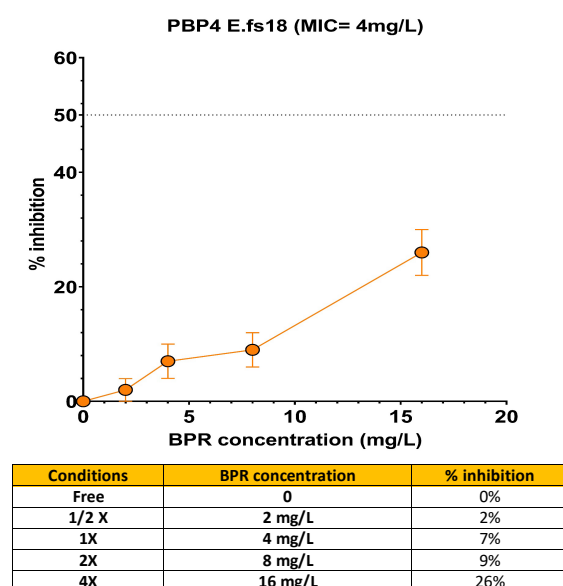

Figure S5. E.fs18 detailed PBPs inhibition rates.

Supplement: Supplementary file 1 [file antibiotics-13-00065-s001.zip › Supplementary material. Figure S5.pdf]
